# Chronic exposure to nicotine in diet enhances the lethal effect of an entomopathogenic fungus in the ant *Cardiocondyla obscurior*

**DOI:** 10.1242/bio.061928

**Published:** 2025-05-06

**Authors:** Jason Rissanen, Dalial Freitak

**Affiliations:** Institute of Biology, Department of Zoology, University of Graz, Graz AT-8010, Austria

**Keywords:** Nicotine, Pollution, Social insect, Ant colony fitness, *Beauveria bassiana*

## Abstract

Nicotine is a naturally occurring alkaloid that has acute toxic effects for insects and affects their behaviour even in sublethal amounts. In addition, nicotine is shown to accumulate and pollute environments through the use of commercially produced pesticides and tobacco products. We investigated how nicotine-polluted diets in two different concentrations impacted colony fitness in the ant *Cardiocondyla obscurior*, compared to a nicotine-free diet. We measured brood production and development, changes in relative abundances of bacterial endosymbionts, and worker survival in combination with a fungal pathogen. Chronic exposure to nicotine caused a concentration-dependent effect in enhancing the lethality of the fungal infection, with higher concentrations causing higher mortality in infected colonies. In the absence of pathogens, nicotine had no effect on worker survival. Furthermore, nicotine did not affect brood production or development, nor clearly affect the abundances of the bacterial endosymbionts. Our results show that nicotine pollution in the environment can negatively affect ant fitness through synergistic effects in combination with a fungal pathogen. Pathogens play a significant part in the decline of insects, and the influence that nicotine pollution may have in exacerbating them should receive more attention.

## Teaser

**Summary:** Nicotine pollution in the environment could have a big impact on the health of ants, as it makes diseases more deadly for them.

## INTRODUCTION

Nutrition is essential not only for survival, but also for fitness of all life and a high-quality food source is a precious commodity. The quality of nutrition can directly affect the growth (Aron et al., 2001; Koyama and Mirth, 2018; Nonacs, 1991), reproduction (Boggs, 1981; Koyama and Mirth, 2018), and immune systems of insects (Garvey et al., 2021; Ponton et al., 2023, 2013). Human impact on ecosystems and the changing climate is altering the nutritional environments of all animals drastically. The use, management and waste of various compounds used by humans for agriculture and industry pollutes the environments and can compromise the quality of available nutrition (Onakpa et al., 2018; Calvo-Agudo et al., 2019, 2022; Zhang et al., 2023). Pesticides such as neonicotinoids, which are still globally the most used pesticide (Simon-Delso et al., 2015), have come under scrutiny due to their wide use and effect on the environment. Neonicotinoids are highly toxic to all insects, and even in sublethal levels affect negatively their behaviour (Galvanho et al., 2013; Sappington, 2018), the composition of gut bacteria (Gong et al., 2023; Sen et al., 2015), as well as exacerbating the effects of pathogen infections (Doublet et al., 2015; Kamiya et al., 2024). Neonicotinoids are picked up by plants from their environments and are present in the nectar and pollen produced by them (Zhang et al., 2023) exposing herbivores, pollinators and other nectar feeders to high concentrations of the pesticides. Additionally, neonicotinoids have also been found in honeydew produced by hemipteran insects in lethal levels for insects (Calvo-Agudo et al., 2019, 2022).

Recently, the role of nicotine pollution in urban environments has received increasing attention. While nicotine is a naturally occurring alkaloid produced by many plants (e.g. in the family *Solenacae*) (Siegmund et al., 1999), the quantity of nicotine in the environment is greatly influenced by human activity through the use of nicotine-based pesticides (Goulson, 2013), as well as the production and careless discarding of tobacco products (Beutel et al., 2021; Ershad et al., 2020; Mohamed, 2023; Roder Green et al., 2014; Selmar et al., 2018). Cigarette butts (CBs) are the most common litter found in urban areas where densities of CBs can spatially be very high (Roder Green et al., 2014; Selmar et al., 2018; Beutel et al., 2021). Several studies have shown that CBs can eluate nicotine and heavy metals into surrounding soil where it can accumulate in high concentrations (Beutel et al., 2021; Dallimore et al., 2021). Nicotine can then further be picked up by plants growing in the polluted soil (Selmar et al., 2015, 2018; Cheng et al., 2021) or be washed into nearby aquatic environments where it can cause toxic effects in the animal communities (Roder Green et al., 2014). Nicotine picked up by plants from their environment is systemically present in the plant matter, and therefore would likely be expressed in the nectar or the honeydew of hemipterans feeding on the plant similarly to neonicotinoids (Calvo-Agudo et al., 2019, 2022), but this is yet to be confirmed empirically. However, it is clear that nicotine has the potential to be a significant pollutant of the environments of wildlife in urban and agricultural areas.

Nicotine is psychoactive molecule, affecting the central nervous system of animals and is historically one of the first commercially produced insecticides due to its acute toxicity in insects (De Ong, 1924). In sublethal concentrations, nicotine has a variety of effects on insect behaviour and cognition, the majority of which have been studied in pollinators, such as honeybees and bumblebees (Thorburn et al., 2015; Baracchi et al., 2017; Mustard et al., 2023), but also in ants (Cammaerts et al., 2014). Nicotine has been shown to affect locomotion (Cammaerts et al., 2014), foraging activity (Cammaerts et al., 2014; Thorburn et al., 2015) as well as memory and cognition (Cammaerts et al., 2014; Thorburn et al., 2015; Baracchi et al., 2017; Mustard et al., 2023) in bees and ants. In terms of health, the consumption of nicotine has been reported to have both medicinal and toxic effects. In bumblebees an increase in nicotine consumption delayed the establishment of the gut parasite *Crithidia bombi* (Baracchi et al., 2015). In honeybees, the presence of nicotine in their diets in low and intermediate concentrations increased survival, but high concentrations of nicotine lowered survival in worker bees (Köhler et al., 2012). Nicotine also have not been found to have any medicinal effects against the bee parasite *Nosema ceranae* (Hendriksma et al., 2020).

Studies on the effects of nicotine on ant health and fitness are severely limited, with most studies focusing on the effects of neonicotinoid pesticides (Schläppi et al., 2020, 2021; Leponiemi et al., 2022). However, due to the chemical similarity between neonicotinoids and nicotine, it is likely that some of the effects on ant health would be similar. Ants are ubiquitous on earth, making up a substantial part of the biomass of insects in ecosystems, as well as playing a key part in their function (Folgarait, 1998; Schultheiss et al., 2022). Ants are known to be diverse in their use of nutrition, exploiting a wide variety of their surroundings varying from scavenging on carcasses and seeds, cultivating fungus, to engaging in mutualistic relationships with plants and insects for nectar and honeydew (Hölldobler and Wilson, 2009). The dietary needs of the colony are influenced by many factors, including size of the colony, the presence of brood (Traniello, 1989; Dussutour and Simpson, 2009), as well as whether the colony is suffering from pathogen infections (Bos et al., 2015; Hsu et al., 2018; Rissanen et al., 2022, 2023; Csata et al., 2024). However, as central place foragers that commonly nest in soil, ants can be particularly susceptible to environmental pollution and are reliant on high-quality nutrition in their nest surroundings. Scavenging on cadavers killed by pesticide exposure or collecting nectar and honeydew from plants and hemipterans growing in contaminated soil can expose ants to lethal amounts of pesticides (Douglas et al., 2015; Calvo-Agudo et al., 2022; Zhang et al., 2023). As nicotine also accumulates in insects and can be picked up by plants from their surrounding soil, ants could be widely exposed to nicotine pollution, yet we know little about the consequences it may have on the fitness of the ant colonies.

In our experiment, we studied how chronic exposure of nicotine-polluted diet affects *Cardiocondyla obscurior* ants in both the presence and absence of a fungal infection. Neonicotinoids have been shown to have a synergistic effect on the lethality of a fungal disease in some ants and termites (Sen et al., 2015; Schläppi et al., 2021) however there are also reports of no synergism between neonicotinoids and pathogens in ants (Leponiemi et al., 2022). Due to nicotine having ant-microbial effects, and being used as a medicinal supplement by bumblebees, our experiment will allow us to study whether nicotine increases or decreases the lethal effect of a fungal infection, depending on whether it has a synergistic or medicinal effect in ants. We measured how different concentrations of nicotine in their diet affect colony fitness metrics in the form of brood production and development. Lastly, we assessed how feeding on nicotine diets affects the relevant abundance of gut symbionts in adult worker ants. To our knowledge, this is the first study on how nicotine affects the colony fitness of ants, as well as the first looking into how it affects the survival of ants in combination with another stressor.

## RESULTS

### Brood

**Fig. 1.**
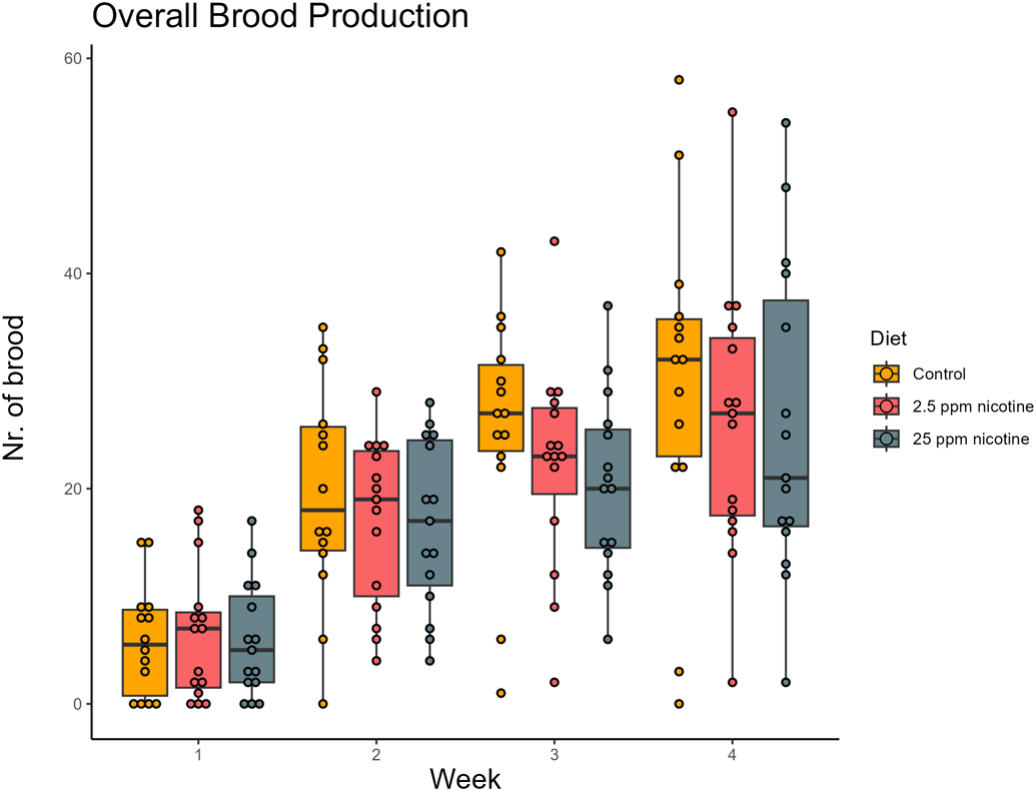
**Overall brood production in colonies of each diet treatment over time (week).** N_Treatment_=30. The amount of brood increased in the colonies for each week (X^2^=76.83, *P*<0.001), but feeding on different concentrations of nicotine in their diet did not alter the brood production in the colonies overall (X^2^=4.121, *P*=0.340).

**Fig. 2.**
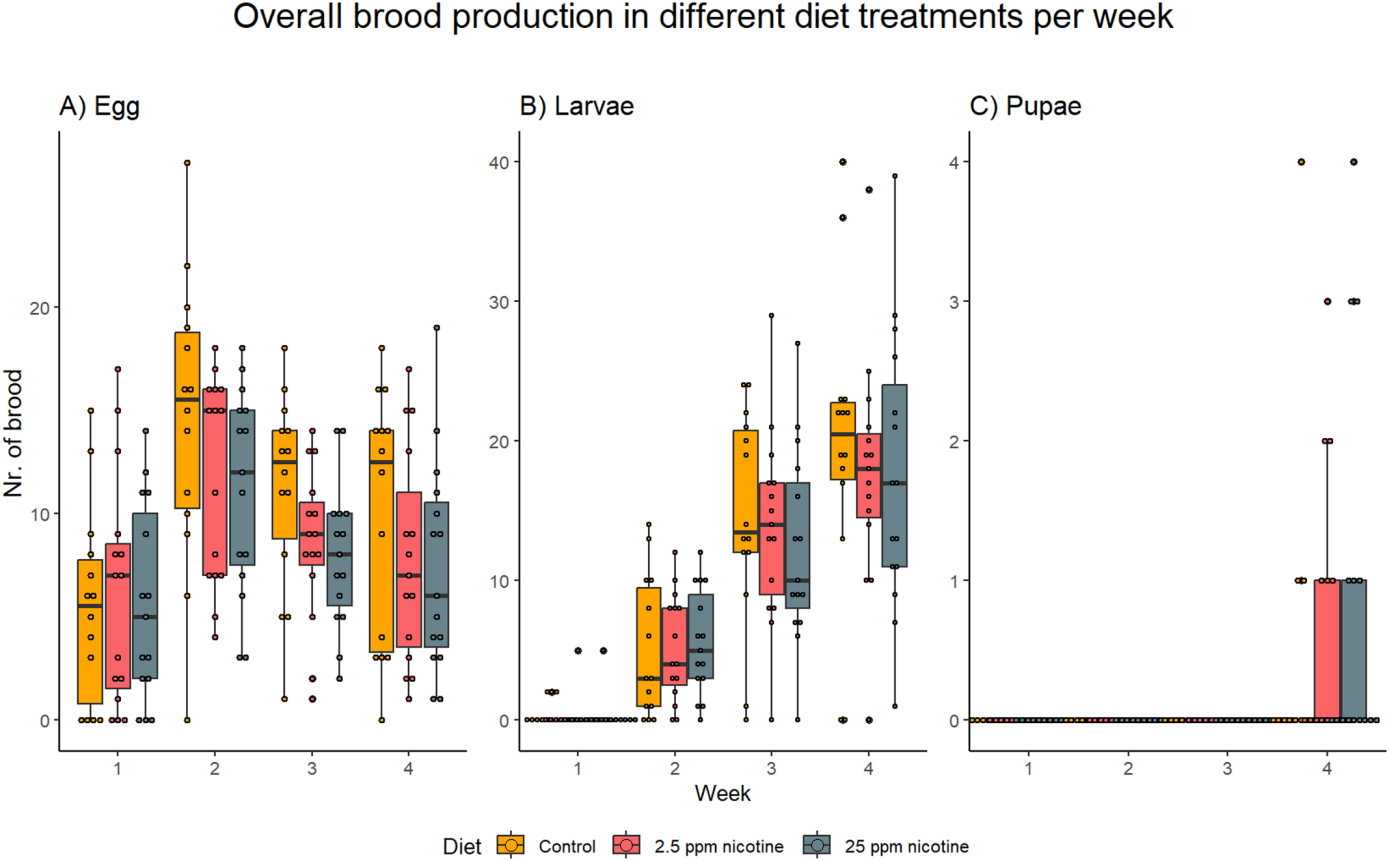
**Brood production of the different brood types, egg (A), larvae (B) and pupae (C) over time in the different diet treatments.** N_Treatment_=30. There was no effect of different nicotine concentrations in the weekly production of brood (X^2^=4.420, *P*=0.620) or the development of the brood (X^2^=3.438, *P*=0.992).

Time significantly affected the amount of brood in the colonies over the 4-week period (week: X^2^=76.83, *P*<0.001), but there was no significant difference in overall brood production caused by the diet (diet: X^2^=4.121, *P*=0.340) (Fig. 1). There was a significant difference in the number of different types of brood in the colonies (brood type: X^2^=133.723, *P*<0.001) and also a difference in the amount of brood of the different brood types during the different weeks, representing brood development over time (brood type×week: X^2^=152.930, *P*<0.001), but the diets caused no significant difference in weekly production nor the development of the different types of brood over time (diet×week: X^2^=4.420, *P*=0.620; diet×brood type×week: X^2^=3.438, *P*=0.992) (Fig. 2).

### Survival

**Fig. 3.**
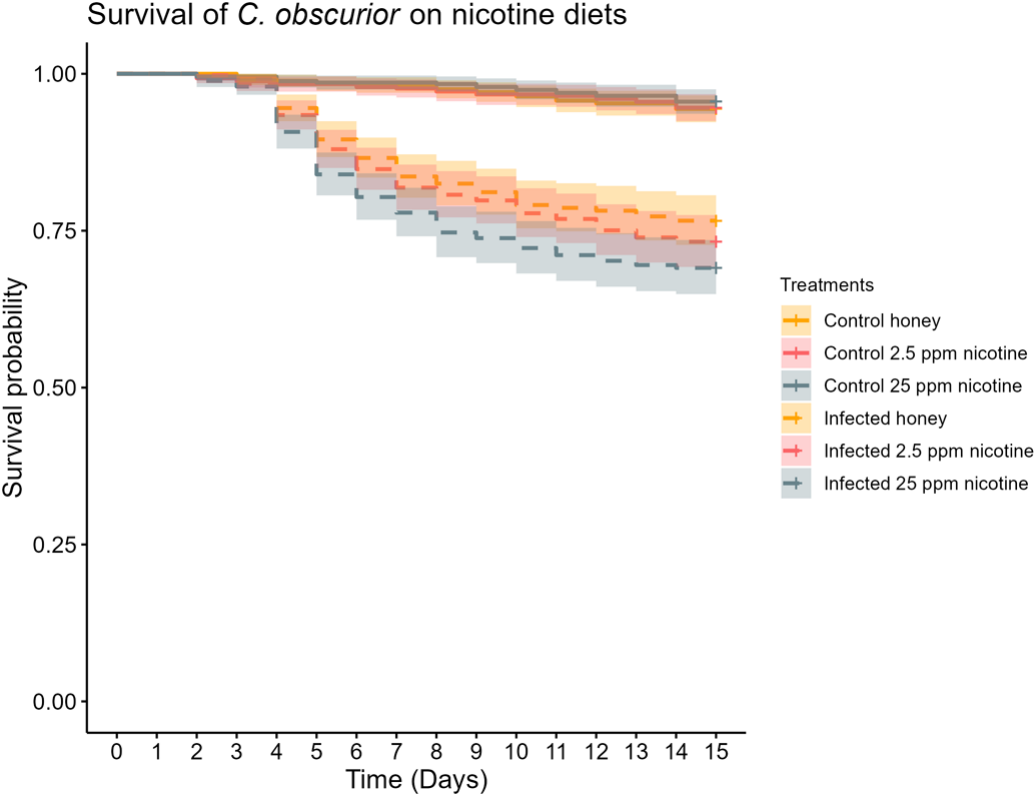
**Survival curve of the colonies of different diet and infection treatments.** N_Treatment_=30. The solid lines represent the sham-treated control colonies, and the dashed lines represent the colonies exposed to the fungal pathogen. Exposure to *B. bassiana* caused significant mortality in the colonies (X^2^=179.901, *P*<0.001), and the colonies feeding on 25 ppm of nicotine experienced significantly higher mortality compared to the colonies feeding on pure honey water (z=−2.626, *P*=0.024). The shaded area represents the 95% confidence interval.

Exposure to the fungal pathogen significantly increased the mortality in the colonies (treatment: X^2^=179.901, *P*<0.001), but the interaction with diet was not significant overall (diet×treatment: X^2^=0.2328, *P*=0.321) (Fig. 3). Within the infected colonies, diet had a significant concentration dependent effect on mortality (X^2^=7.010, *P*=0.030), where the high-nicotine colonies were suffering significantly higher mortality than the control-diet colonies (z=−2.626, *P*=0.024) but the low-nicotine diet colonies were significantly not surviving differently from either the control diet colonies (z=−1.119, *P*=0.502), or the high nicotine diet colonies (z=−1.524, *P*=0.280). Within the control treatment colonies, there was no difference in mortality caused by diet (control versus low concentration: z=0.125, *P*=0.994; control versus high concentration: z=0.777, *P*=0.717; low concentration versus high concentration: z=0.646, *P*=0.794).

### Bacterial endosymbionts

**Fig. 4.**
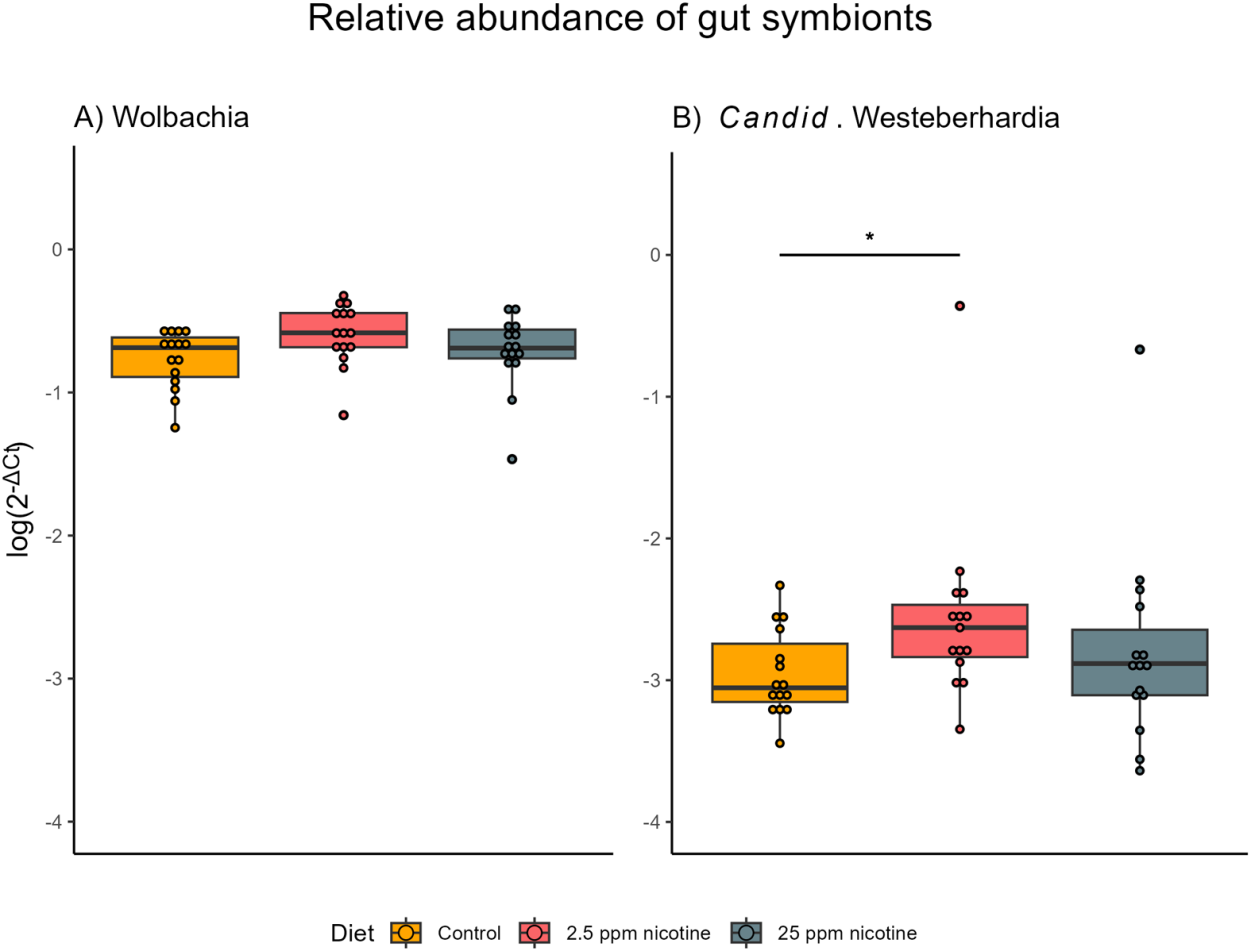
**Relative abundance of the gut symbionts Wolbachia (A) and *Candid.* Westeberhardia (B) in the different diet treatments.** N_Treatment_=15. There was a significant decrease of the relative density of *Candid*. Westeberhardia in colonies on the 2.5 ppm diet compared to the control diet colonies (t=2.532, *P*=0.036), but no effect of other diets.

The ants overall contained a higher relative abundance of *Wolbachia* over *Candid.* Westeberhardia regardless of diet (gene×diet: *F*=0.487, *P*=0.616) (Fig. 4). Diets did have an overall effect on the relative abundance of the gut symbionts (diet: *F*=3.143, *P*=0.048). Pairwise comparisons showed no significant difference in the abundance of *Wolbachia* between the diets (control versus low concentration: t=−1.089, *P*=0.524; control versus high concentration: t=−0.323, *P*=0.944; low concentration versus high concentration: t=0.766, *P*=0.725). There was a significant decrease in the amount of *Candid.* Westeberhardia in the 2.5 ppm diet compared to the control diet (2.5 versus honey: t=2.532, *P*=0.036). The other comparisons showed no significant differences (control versus high concentration: t=−0.967, *P*=0.600; low concentration versus high concentration: t=1.564, *P*=0.268).

## DISCUSSION

Due to the toxicity of nicotine, we expected to see an effect on ant survival caused by the concentration of nicotine even in the absence of another stressor. Our colonies were exposed to the different nicotine diets for a total of 6 weeks, which is a substantial amount of time in combination with high nicotine concentrations, yet we found no evidence for a direct toxic effect in ants in terms of survival over this time. It is possible that a longer exposure could prove a direct toxic effect for ants, but in our experiment, we found no trend towards this effect. We also found no evidence of a concentration-dependent effect of ingesting nicotine on the relative amount of obligate gut symbionts, something that has been shown in this ant species before with chronic exposure to the herbicide glyphosate in the diet of the ants (Leponiemi et al., 2022). However, we observed that ants chronically exposed to nicotine in their diet suffer higher mortality when faced with an additional stressor, a pathogenic fungus. This effect is clearly concentration dependent, with colonies feeding on a diet of higher concentrations suffering higher mortality than those feeding on a lower concentration or in the absence of nicotine in their diet.

While nicotine has been reported to have medicinal benefits against pathogens in honeybees (Köhler et al., 2012) bumblebees (Baracchi et al., 2015) and moth larvae (Garvey et al., 2021), we found no evidence that the *C. obscurior* ants were able to use nicotine in diet against a fungal infection. Quite the opposite, the fungal infection was more severe as the concentration of nicotine was higher in their diets. The enhancement of a fungal infection has been reported in some ants in combination with neonicotinoids (Schläppi et al., 2020), but there are also conflicting reports on no synergistic effect in *C. obscurior* (Leponiemi et al., 2022). Our result shows that the synergistic effect is also apparent with nicotine polluted diets. The mechanisms of the synergistic effect remain unknown from our experiment, as we collected limited physiological data. Neonicotinoids have been shown to suppress immune responses in insects (Doublet et al., 2015; Schläppi et al., 2020, 2021; Kamiya et al., 2024) as well as affecting hygienic behaviours in ants (Galvanho et al., 2013), which could further enhance the effect of a fungal pathogen. The effect of nicotine in these regards is not known in ants, and future studies should take a detailed look into how nicotine causes a higher mortality when in combination with a fungal pathogen.

Exposure to nicotine did not affect any of the colony fitness metrics we measured in our experiment. Brood production and brood development were not affected by the presence of nicotine in the diet, even if it has been reported with an exposure to high concentration nicotine diets in honeybees (Köhler et al., 2012). Developing brood feed mostly on protein (Hölldobler and Wilson, 2009), which in our experiment was untainted by nicotine, which could explain why the developing brood did not differ in the different treatments if they were not exposed to nicotine. A similar study with *C. obscurior* using neonicotinoid and glyphosate polluted foods saw a similar result regarding no significant effect on brood production or brood development (Leponiemi et al., 2022).

We measured how the differently polluted diets would affect the relative abundance of gut symbionts to see whether nicotine could have a direct effect on the overall gut microbiome in ants, but we found no evidence of a clear effect on gut symbionts that would reflect the effect on mortality when feeding on nicotine diets. There was only a significant effect on the abundance of *Candid.* Westeberhardia in the colonies with the intermediate concentration of nicotine in their diets (2.5 ppm), where the colonies had a slight reduction of the gut symbionts. While nicotine does seem to have a minor effect on the gut microbiome of ants, it is likely that the effect on the abundance of *Candid.* Westeberhardia is not linked to the increased mortality due to the pathogen, as the effect on the gut symbionts was not evident in the highest concentration of nicotine in the diet where mortality was the highest. There could be several reasons why an intermediate nicotine content in the diet influences the abundance of *Candid*. Westeberhardia. However, in light of the results and the limitations of our experimental setup to study causality of gut symbiont change due to nicotine diets, this would be speculative at best, and it should rather be studied in a more detailed manner in the future.

Our result highlights the importance of studying combinations of stressors and the interplay between them. Solely looking at the seemingly non-existent effects of nicotine on the fitness of ants might give the impression that nicotine bears no harm to them, as it does not affect individuals or the productivity of their colonies. However, the effect of nicotine is only apparent when ants are exposed to a pathogenic fungus, and the effect is clear with colonies feeding on a high concentration of nicotine suffering a higher mortality compared to colonies feeding on just honey water. While many ants are generalists regarding their nutrition and can forage in wide areas around their nests, having a centralized nest means they are restricted to a fixed area. Relocation to other areas in the search for a better nutritional environment can be impossible or extremely costly for large colonies of ants. Therefore, any compromise of the surrounding sources of food can further affect the fitness of entire colonies.

Neonicotinoids are, for good reason, garnering a lot of attention due to their high toxicity and wide susceptibility of insects to them. The mounting evidence on unwanted side-effects of the prolific use of neonicotinoids on environmental pollution and the subsequent health of animals, including humans, has led to widespread regulation of the use of them. Nicotine, however, remains neglected and severely understudied as an environmental pollutant, regardless of how prevalent nicotine waste is in our environments. The role of nicotine pollution in our environments, both urban and agricultural, is a vital issue for the health and wellbeing of our wildlife. Nicotine waste and its pollution will remain a considerable threat to animals, and we need to study the effect of it in combination with other stressors in the form of other pollutants, changing climate, and pathogens.

## MATERIAL AND METHODS

### Study organisms

*Cardiocondyla obscurior* is an arboreal species of ants originating from tropical south-east Asia but has spread throughout the tropics in the rest of the world. Colony sizes range from dozens of workers and a single queen to hundreds of workers and dozens of queens. The primary method of dispersal of *C. obscurior* is through the budding of workers and queens when the colony grows large, making them a great laboratory study system (Heinze et al., 2006). We used laboratory reared colonies, obtained from the university of Regensburg, which were originally collected in Japan in 2010.

*Beauveria bassiana* is a generalist entomopathogenic fungus present in soils all around the world, and is a common pathogen used in studying pathogen effects in ants (Oi et al., 1994; Malagocka et al., 2019; Leponiemi et al., 2022; Rissanen et al., 2023). We grew *B. bassiana* (strain IMB 7389) on solid malt-peptone agar plates in the dark at room temperature.

Nicotine (Sigma-Aldrich, N0267) was in liquid form and further diluted in 75% (w/v) honey-water solution to the desired concentrations for the use in the experiment.

### Experimental colonies

We used 15 original lab-reared nests as stock colonies and used each one to make six experimental colonies, each to be used in different treatments to minimize any nest-caused differences (three different diets, with and without subsequent fungal infection). To each experimental colony we counted 33 workers and two queens. Each experimental colony was placed into separate nest box made of Petri-dishes with plaster covering half of the base of the dishes. An indentation was cast in the plaster to function as a nesting site for the colonies, with a strip of tinfoil for cover. Each colony was provided with water in Wettex strips and 30 µl of a 75% honey-water solution (Steirishces Imkerzentrum: wildflower honey) containing different concentrations of nicotine on a small paper strip square. Water and food were replaced three times a week (Monday, Wednesday, Friday). Both water and food were placed on small metal discs and placed in the colonies on the side not covered with plaster for hygienic upkeep. The colonies were also given a protein source three times a week: on Mondays and Wednesdays the colonies were given a piece of desert locust (*Schistocerca gregaria*) thorax, and on Fridays 10 fruit flies (*Drosophila melanogaster*). The amount of honey-water solution and protein provided was never completely consumed, therefore considered to be an *ad libitum* amount of nutrition. The colonies were kept in an incubator with a 12:12 h light/dark cycle in a stable environment of 25°C temperature and 60% relative humidity.

In parallel to the experimental colonies, we established a further three colonies, one for each diet treatment, from each original nest to function as stock colonies for replenishing workers and queens in the experimental colonies to counteract handling mortality caused by establishment of the experimental colonies.

### Diet treatments

To study the effects of nicotine on the fitness of the colonies both pre- and post-infection, we split the colonies into three different diet treatments: control food (75% honey-water), low concentration of nicotine (2.5 ppm in 75% honey-water), high concentration of nicotine (25 ppm in 75% honey-water). 2.5 ppm of nicotine is representative of nicotine concentrations in nature, and has been used in other studies as a naturally relevant concentration, as plants in the Nicotiana family having concentrations of nicotine in nectar varying to up to 5 ppm (Baracchi et al., 2015, 2017). 25 ppm of nicotine is higher than found in plant nectar, but relevant in consideration to nicotine pollution from tobacco farming and discarding cigarette butts in nature which can accumulate in soils in similar and higher concentrations (Dallimore et al., 2021).

For 4 weeks, the colonies were given their assigned diets, 30 experimental colonies on each diet for a total of 90 colonies (*N*=30+30+30=90). All colonies were observed to have foragers feeding on the honey solutions, but the amount of consumption was not quantified. Over the 4 weeks, we observed mortality, as well as assessing brood production by counting eggs produced and their development to larvae and pupae in the colonies once a week (Friday). Any ants that died, queens or workers, were replaced from stock colonies made from the initial nests that had been given the same diet as their experimental colonies.

### Infection treatment

After 4 weeks, half of the colonies from each diet treatment were exposed to conidia of the fungal pathogen, while the rest were sham-treated controls in the way that the original nests were represented in each treatment for each diet. Conidia of *B. bassiana* were collected from two plates by pouring 10 ml of 0.05% Triton-X onto the plate and carefully rubbing the surface of the fungus with a sterile glass rod to harvest the conidia. The conidia suspensions were centrifuged at 3000 rpm for 3 min, after which the supernatant was discarded, and the conidia resuspended in 5 ml of 0.05% Triton-X. The conidia solutions from the two different plates were then combined. The concentration of conidia was determined with a hemocytometer (Bürker) and then diluted to the desired concentration of 1×10^8^ conidia ml^−1^ in a 0.05% Triton-X solution. For the infections, we exposed the ants to 5 s of CO_2_ gas to anesthetize them and placed them on an absorbent tissue paper. We then pipetted 5 µl of the conidia solution onto each individual ant, after which returning them to their nest boxes. The control colonies were treated identically apart from being pipetted with clean 0.05% Triton-X. The queens were captured and isolated during this time and did not undergo anesthetization or infection treatments.

After the infection treatment, each treatment consisted of 15 colonies (infected: *N*=15+15+15=30, control: *N*=15+15+15=30), each with 30 workers and two queens each after sampling for the gut symbiont analysis. For the next 2 weeks, we performed the feeding according to the same protocol as during the pre-infection phase, however now mortality in colonies was monitored daily and dead ants were removed from the colonies but not replaced. During the infection phase we did not monitor brood production, as there was a potential loss or damaging of the brood during the infection treatment, however we did remove pupae from the colonies to make sure the number of workers in the colonies was not altered due to brood development into new adults.

### Bacterial endosymbionts

To study how the exposure of nicotine affects the relative abundance of two gut symbionts in *C. obscurior*, we collected samples of adult workers from each colony after 4 weeks of exposure to different diets. We collected three ants from both experimental colonies that shared the original nest that were assigned to different infection treatments (infected / control, *N*=3+3=6 ants). All samples were stored at −20°C for further processing. We extracted genomic DNA from whole ants using the QIAmp DNA micro kit (Qiagen) by crushing the whole ants with a sterile pestle, and processed further according to the manufacturers protocol, eluting the isolated DNA into 45 µl of the provided elution buffer for PCR amplification.

The two bacterial gut symbionts we assessed were *Candidatus* Westeberhardia cardiocondylae and *Wolbachia*. Both gut symbionts are considered to be in obligate symbiosis with host and have the potential to affect life-history traits in the ants and possibly the immune system as well (Klein et al., 2016; Ün et al., 2021), and have been studied before in studies exposing ants to pesticide polluted diets (Leponiemi et al., 2022). *Candid.* Westeberhardia contributes to the synthesis of tyrosine and contributes to host cuticle formation (Klein et al., 2016). *Wolbachia* can influence the reproduction of its hosts through the induction of cytoplasmic incompatibility (Ün et al., 2021). For the quantification of Wolbachia levels in the ants, we used primers specific for the cytochrome oxidase A gene (Ün et al., 2021). *Candidatus* Westeberhardia levels were quantified using primers for the nrdb gene (Klein et al., 2016). The elongation factor 1-alpha 1 (EF1) gene was used as a reference gene.

The relative abundance of gut symbiont densities was determined using Real-Time quantitative polymerase chain reaction (RT-qPCR) using the CFX384 Real-Time System (Biorad) in 384-well hard-shell PCR plates. The samples were analyzed using SsoAdvanved™ Universal SYBR^®^ Green Supermix (Biorad) with a final volume of 20 µl [14 µl master mix: 10 µl SYBR+2 µl forward primer+2 µl reverse primer; 6 µl sample (4 ng µl^−1^ DNA)]. We used two technical replicates for each sample. The pcr was set for a cycle program of 1×95°C 3 min; 40×95°C 5 s, 60°C 20 s; 95°C 10 s.

### Statistical analysis

All data were analyzed using the R software (version 4.3.0) and all plots were drawn with ggplot2.

The brood production was analyzed using generalized linear mixed-models using the glmmTMB function from the *glmmTMB* package (Brooks et al., 2017). In the model we used the number of brood (brood types: eggs, larvae, pupae) as the response variable with brood type, week (1–4), and diet (control, low nicotine, high nicotine) as well as their interaction as fixed-factors using a negative binomial distribution. The colony ID was added as a random factor as we did repeated counts of the same colonies [brood∼diet×brood type×week+(1|colony)]. The significance of the fixed effects and their interaction in the model was determined using an ANOVA.

The survival data were analyzed with a cox proportional hazard model using the coxme function from the *coxme* package (Therneau, 2024). We excluded any mortality that occurred in the first day after treatment from the analysis, as it was likely due to handling stress rather than mortality caused by the pathogen. The omission of the handling mortality did not significantly alter the results. In the model, we used the diet and treatment as fixed factor as well as their interaction, with the colony as a random factor to account for pseudoreplication [(day, survival) ∼diet×treatment+(1|colony)]. The significance of the fixed effects and their interaction in the model was determined using an ANOVA from the car package. Pairwise comparisons were conducted using the emmeans function from the *emmeans* package (Lenth, 2022.) with a Tukey's *P*-value correction.

The relative abundance of Wolbachia and *Candid.* Westeberhardia were analyzed with a generalized linear model using the glm function in the lme4 package (Bates et al., 2015). We normalized the threshold cycles values to the housekeeping gene EF1 (ΔCt) and transformed them to log(2^−ΔCt^) for clarity and used the values as the response variable in the model. The interaction of diet and gene were used as the fixed factors (model formula: log(2^−ΔCt^)∼diet×gene). The significance of the factors and their interaction was determined with an ANOVA of the model. Pairwise comparisons were conducted using the emmeans function as described above.
